# Elevated plasma p-tau217 in newborns: developmental signal or synaptic stress marker?

**DOI:** 10.1093/braincomms/fcaf335

**Published:** 2025-09-04

**Authors:** Caterina Motta, Chiara Giuseppina Bonomi, Alessandro Martorana

**Affiliations:** Memory Clinic and Neurodegenerative Dementia Research Unit, Policlinico Tor Vergata, University of Rome ‘Tor Vergata’—viale Oxford 81, Rome 00133, Italy; Memory Clinic and Neurodegenerative Dementia Research Unit, Policlinico Tor Vergata, University of Rome ‘Tor Vergata’—viale Oxford 81, Rome 00133, Italy; Memory Clinic and Neurodegenerative Dementia Research Unit, Policlinico Tor Vergata, University of Rome ‘Tor Vergata’—viale Oxford 81, Rome 00133, Italy

## Abstract

The finding of elevated plasma p-tau217 in newborns raises the question of whether it represents a developmental signal or an early marker of synaptic stress, offering new insights into the biology of tau in the human brain.

The recent article ‘The potential dual role of tau phosphorylation: plasma phosphorylated-tau217 in newborns and Alzheimer’s disease’^1^ in *Brain Communications* reports that levels of phosphorylated tau at threonine 217 (p-tau217) in newborns are markedly higher than those observed in patients with Alzheimer’s disease, supporting the intriguing hypothesis that phosphorylation of tau may serve a physiological role in early life distinct from its pathological aggregation in neurodegenerative disease. This concept is potentially paradigm-shifting, inviting a re-evaluation of the canonical view of phosphorylated tau as exclusively a marker of neurodegeneration.

Indeed, phosphorylation of tau modulates its binding to microtubules and has been implicated in normal neuronal signaling and cytoskeletal dynamics^2^ but also in the development of tauopathies when dysregulated.^3^.

In the context of early brain development, several mechanisms may contribute to elevated tau levels in the newborn period. One of these is postnatal apoptosis, a well-documented physiological process in animal models, which eliminates excess or improperly connected neurons to refine neural circuits.^4^ Remarkably, although up to 30% of cortical neurons may be lost during this phase, brain volume continues to increase, largely due to glial proliferation and the expansion of the neuropil.^5^ This intense remodelling of the developing brain may result in the transient release of tau protein into the cerebrospinal fluid and blood, reflecting active synaptic reorganization rather than pathology.

However, while the authors propose a role for p-tau217 in healthy neurodevelopment, their own longitudinal data reveal a rapid normalization of plasma p-tau217 levels within few weeks after birth in a subgroup of neonates.^1^ This temporal profile challenges the interpretation of p-tau217 as a marker of sustained developmental plasticity. If phosphorylated tau played a beneficial role in early brain development, its levels would be expected to remain elevated throughout the key periods of synaptic plasticity in infancy, rather than showing such a rapid postnatal decline. This apparent inconsistency prompts consideration of alternative explanations for the neonatal surge in plasma p-tau217 levels.

One hypothesis worth exploring is that the elevated plasma p-tau217 in neonates may not indicate a physiological role in neurodevelopment per se but rather a transient response to perinatal stress. The process of birth itself—even in uneventful deliveries—imposes significant mechanical and metabolic challenges on the neonatal brain, possibly including brief episodes of hypoxia. Moreover, the immediate postnatal period following premature birth is often marked by haemodynamic instability, which can lead to tissue hypoperfusion.^6^

This leads us to consider whether the observed increase in p-tau217 mirrors changes seen in different hypoxic neurological conditions. Interestingly, in patients resuscitated from cardiac arrest, plasma levels of p-tau231 peaked on hospital admission and gradually decreased between admission and 12 to 48 h, suggesting a direct link between acute cerebral stress and tau release into the bloodstream.^7^ Similarly, in the context of ischaemic stroke, elevated plasma tau concentrations have been observed in the acute phase, correlating with the extension of neuronal injury.^8^

These findings suggest that the high levels of p-tau217 observed in neonates could reflect a transient injury-like response—perhaps a form of ‘synaptic stress remodelling’—rather than a purely developmental function. This interpretation aligns with the idea that tau phosphorylation may play a broader role in synaptic homeostasis under stress, whether that stress is chronic, as in Alzheimer’s disease, or transient, as during birth. Such a model would reconcile the shared biomarker with differing underlying mechanisms. In Alzheimer’s disease, persistent p-tau accumulation signals ongoing pathological remodelling and eventual synaptic failure, while in neonates, transient elevation may reflect a reversible and adaptive process in response to a stressful transition to extrauterine life.

It remains possible that both mechanisms co-exist: tau phosphorylation might be engaged in neurodevelopmental plasticity but become dysregulated in the context of injury or disease. However, the rapid normalization of p-tau217 levels in neonates suggests a narrow temporal window misaligned with a sustained developmental role.

In conclusion, while the authors’ hypothesis regarding the beneficial role of p-tau217 in neonatal plasticity is compelling,^1^ alternative interpretations should be considered. The available data—including the temporal dynamics of p-tau217 normalization and parallel evidence from studies of acute brain injury—support the possibility that p-tau217 in newborns may signal early-life synaptic stress rather than physiological plasticity alone.

This hypothesis could open new avenues for research aimed at understanding how the neonatal brain rapidly restores tau homeostasis. Investigating the molecular and cellular mechanisms that allow for this prompt normalization of p-tau could provide valuable insight into how synaptic resilience is achieved in early life, and may ultimately inspire novel therapeutic strategies aimed at enhancing synaptic recovery in Alzheimer’s disease.

## Data Availability

There are no new data associated with this article.
